# Upregulation of long noncoding RNA HOTTIP promotes metastasis of esophageal squamous cell carcinoma via induction of EMT

**DOI:** 10.18632/oncotarget.12995

**Published:** 2016-10-31

**Authors:** Xuemei Chen, Hongyu Han, Yuqi Li, Qiongxia Zhang, Kailan Mo, Size Chen

**Affiliations:** ^1^ School of Public Health, Southern Medical University, Guangzhou, 510515, China; ^2^ Cancer Center, Sun Yat-Sen University, Guangzhou, 510080, China; ^3^ Department of Oncology, The First Affiliated Hospital of Guangdong Pharmaceutical University, Guangzhou, 510080, China

**Keywords:** HOTTIP, long noncoding RNA, ESCC, proliferation, EMT

## Abstract

Esophageal squamous cell carcinoma (ESCC) is one of the prevalent and deadly cancers worldwide, especially in Eastern Asia. The prognosis of ESCC remains poor; thus, it is still necessary to further dissect the underlying mechanisms and explore therapeutic targets of ESCC. Recent studies show that lncRNAs involve in the initiation and progression of various cancers including ESCC. HOTTIP has been recently revealed as oncogenic regulator in different cancers, however, whether HOTTIP is involved in ESCC remains poorly understood. To investigate the role of HOTTIP in ESCC, we evaluated the HOTTIP expression levels in a series of ESCC tissues and a panel of ESCC cell line using qRT-PCR. Moreover, we investigated the effect of HOTTIP on cell proliferation, migration and invasion of ESCC cells. Here, we reported that HOTTIP was upregulated in ESCC. Further experiments revealed that HOTTIP knockdown significantly inhibited ESCC cells proliferation by causing G1 arrest. Furthermore, inhibitory effects of HOTTIP on cell migration and invasion were partly associated with EMT process. In conclusion, these data suggest that HOTTIP could be an oncogene for ESCC, and may be served as a candidate target for new therapies in human ESCC.

## INTRODUCTION

Esophageal cancer is considered as a common carcinoma as well as the sixth frequent cause of death all over the world [1]. Esophageal squamous cell carcinoma (ESCC) is the primary subtype, which arised from esophageal epithelial cells [2]. Clinically, individuals with stage III ESCC usually have poor prognosis, with overall five-year survival of approximately 10–15%, and the median survival for late stage patients is less than 12 months [3]. Cancer is widely regarded as a genetic disease, and ESCC is no exception. However, the molecular and genetic basis of esophageal carcinogenesis has not been clearly elucidated. The prognosis of ESCC remains unsatisfactory because the understanding of the molecular mechanisms of ESCC development and progression is currently not available. Therefore, it is critical to identify new biomarkers and therapeutic targets to improve ESCC diagnosis and treatment.

Long non-coding RNAs (lncRNAs), a new class of non-coding RNAs with > 200 bases, have limited or lack protein-coding capacity [4]. LncRNAs are recently increasingly emerging as molecules that take its part in human carcinogenesis [5]. LncRNAs also function as a molecular sponge for miRNAs to antagonized its target mRNA expression [6–7]. In past 3 years, accumulating researches about lncRNAs have emerged in ESCC field. The lncRNA AFAP1-AS1 is upregulated in esophageal adenocarcinoma, and *in vitro* experiments showed that AFAP1-AS1 promotes invasion and metastasis. Although a decade of research contributed to better understand lncRNAs functions, only a few have been designated. Indeed, most lncRNAs remain largely unknown, especially concerning ESCC. Recently, increasing evidence has shown that HOXA transcript at the distal tip (HOTTIP), situated at the 5′ end of the HOXA cluster, was shown to be dysregulated in various cancer [8]. The activity of HOTTIP is the consequence of its interaction with the WDR5/MLL complex, which promotes histone H3 lysine 4 trimethylation to upregulate multiple 5′ HOXA genes expression [9]. However, its expression, roles, and functions in ESCC are still elusive and need to be investigated deeply t. The aim of this study was to identify the role of HOTTIP in the regulation of ESCC progression and pathogenesis.

## RESULTS

### The expression of lncRNA HOTTIP is upregulated in ESCC tissues and cell lines

**Figure 1:**
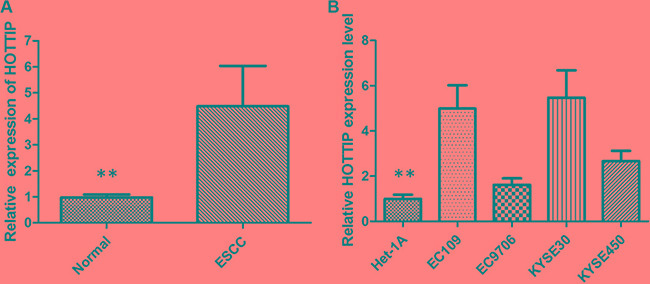
(**A**) HOTTIP was detected in ESCC tissues and adjacent noncancerous tissues by qRT-PCR; (**B**) qRT-PCR showing expression level of HOTTIP in ESCC cell lines.

The expression of HOTTIP was examined by qRT-PCR in 78 pairs of cancerous and the corresponding adjacent noncancerous tissues that were from ESCC patients. The relative expression of HOTTIP in ESCC tissues compared with noncancerous tissues is shown in Figure 1A. Compared with normal tissue, the HOTTIP expression level was significantly increased in 64.10% of ESCC tissue samples (50/78). Furthermore, elevated HOTTIP expression level was predominantly found in late-stage tumor tissues and positively correlated with tumor size. The expression of HOTTIP was not correlated with other clinical factors such as age and location. Then qRT-PCR for HOTTIP was performed in a panel of ESCC cell lines and the expression level of HOTTIP was upregulated in all ESCC cells when normalized to Het-1A (Figure 1B). We found HOTTIP was most upregulated in EC109 and KYSE30 cells; however, EC9706 cells showed lower expression of HOTTIP. Therefore, EC109, KYSE30 and EC9706 were selected as our experimental cell lines.

### HOTTIP mediated cell growth and cell cycle of ESCC cells

**Figure 2:**
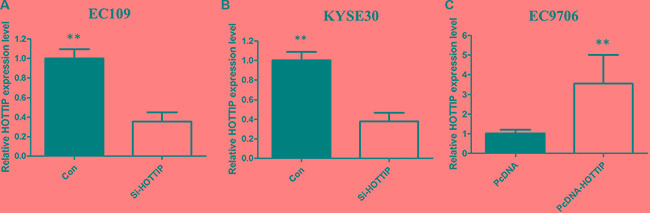
We employed siRNA and expressing plasmid to enhance efficiency of HOTTIP knockdown and overexpression in ESCC cell lines

**Figure 3:**
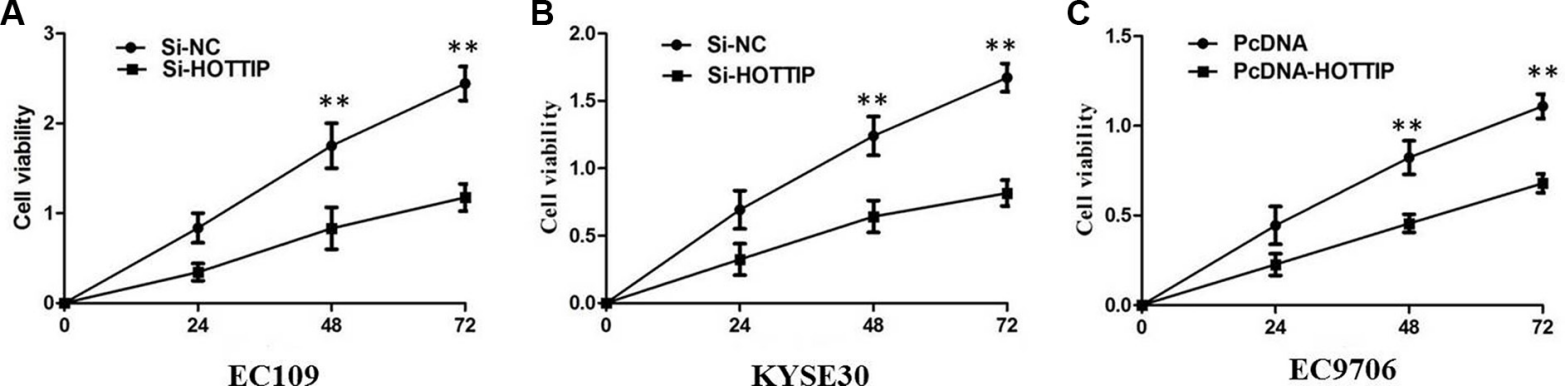
(**A**) CCK8 assay showing knockdown of HOTTIP inhibited cell proliferation of EC109 cells. (**B**) CCK8 assay showing knockdown of HOTTIP inhibited cell proliferation of KYSE30 cells; (**C**) CCK8 assay showing overexpreesion of HOTTIP promoted cell proliferation of EC9706 cells.

**Figure 4:**
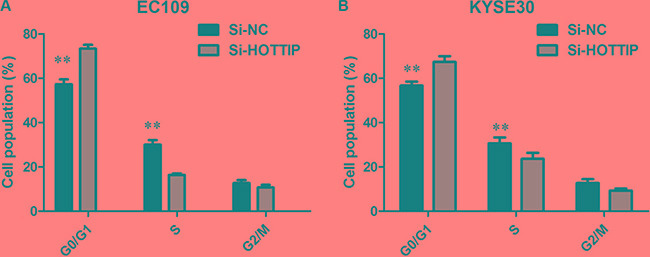
(**A**) EC109 cells transfected with si-HOTTIP all had cell-cycle arrest at the G1-G0 phase compared with cells transfected with si-NC; (**B**) KYSE30 cells transfected with si-HOTTIP had cell-cycle arrest at the G1-G0 phase compared with cells transfected with si-NC.

To further investigate the roles of HOTTIP on regulating ESCC cell phenotypes, and mechanism investigations document by which mechanism HOTTIP regulating its underlying targets, *in vitro* loss- and gain-of function assays were performed. We employed siRNA and expressing plasmid to enhance efficiency of HOTTIP knockdown and overexpression in ESCC cell lines (Figure 2A–2C). The CCK-8 assay results showed that HOTTIP downregulation significantly impeded the proliferation of EC109 and KYSE30 cell lines, and overexpression of HOTTIP increased the ability of cell proliferation of EC9706 (Figure 3A–3C). We then performed flow cytometric analyses to further evaluate whether HOTTIP plays a role in ESCC cell cycle to affects proliferation. Suppression of HOTTIP decreased the S-phase pencentage and increased G0/G1 phase percentage of EC109 and KYSE30 cells (Figure 4A and 4B).

### HOTTIP regulates ESCC cell migration and invasion via induction of EMT

**Figure 5:**
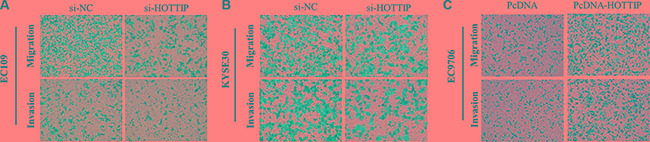
(**A**) Inhibition of Migration and Invasion of EC109cells by HOTTIP siRNA; (**B**) Inhibition of Migration and Invasion of KYSE30 cells by HOTTIP siRNA; (**C**) Overexpreesion of HOTTIP promoted Migration and Invasion of EC9706 cells.

**Figure 6:**
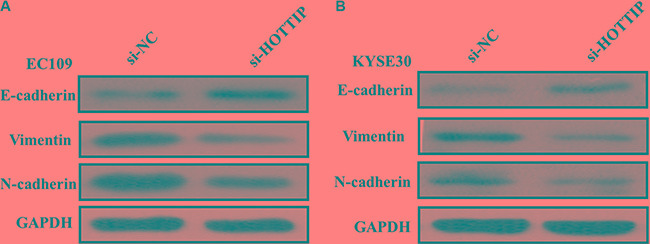
(**A**) Knockdown of HOTTIP reverses EMT in EC109 cells; (**B**) Knockdown of HOTTIP reverses EMT in KYSE30 cells.

Next we identified the effect of HOTTIP on invasiveness of ESCC cells. We found that HOTTIP inhibition significantly decreased the migration and invasion capability of EC109 and KYSE30 cells (Figure 5A and 5B). Conversely, the migration activity of HOTTIP-overexpressing EC9706 cells was significantly increased (Figure 5C). Because EMT is the remarkable presentation for cell invasion, whether silencing HOTTIP expression inhibited mesenchymal features need to be identified. As showed in Figure 6, Vimentin and N-cadherin were downregulated after HOTTIP knockdown while E-cadherin was overexpressed in EC109 and KYSE30 cells (Figure 6A and 6B). Therefore, inhibition of HOTTIP in ESCC cells makes the cell phenotype be more epithelial rather than mesenchymal.

## DISCUSSION

ESCC is the leading prevalent histopathologic subclass of esophageal cancer in central regions of China [10]. Despite of the recent rapid promotion in the diagnosis and therapy, the prognosis of ESCC remains poor [11]. The improvement of ESCC survival rate requires a clear understanding of pivotal molecular mechanisms from the initiation and progression of ESCC. LncRNAs have been shown to aberrantly express in a variety of cancers including ESCC [12–13]. Specific lncRNAs have been shown to promote the development and progression of ESCC. Increased HOTTIP expression has been reported in lung cancer, pancreatic cancer, and hepatocellular carcinoma [14–15]. Among these cancers, HOTTIP might regarded as an oncogene, and HOTTIP overexpression was correlated to enhanced cell proliferation, migration but reduced apoptosis [16]. Nevertheless, the functions of HOTTIP in ESCC were previously unexplored. Thus in current study, we showed that HOTTIP was significantly upregulated in ESCC tissues and cell lines, meaning that elevated HOTTIP expression was positively associated with ESCC carcinogenesis.

Previous study reported that silencing HOTTIP attenuated hepatocellular carcinoma cell proliferation *in vitro* as well as tumorigenicity *in vivo*. To further understand the mechanism of HOTTIP in ESCC progression, *in vitro* loss- and gain-of function assays were conducted. We found that knockdown HOTTIP could inhibit the capability of ESCC cell proliferation compared with control, suggesting that increased HOTTIP expression could promote the ESCC progression. Moreover, depletion of HOTTIP led to G0/G1 phase cell cycle arrest.

Metastasis is the main cause of mortality in cancer patients. Previous studies showed that in colorectal cancer cells, HOTTIP knockdown also inhibited migratory capability and significantly decreased lung metastatic lesions in mouse xenograft mode, similar to the results in our study. We found that knockdown of HOTTIP also inhibited migratory ability of ESCC cells. EMT, an important process that is associated with the progression and metastasis, was also observed in ESCC. Whether HOTTIP plays a role in EMT has not been reported till now. In our study, HOTTIP knockdown downregulated Vimentin and N-cadherin and upregulated E-cadherin. These findings pointed out that HOTTIP might function as an oncogene that was involved in ESCC carcinogenesis and HOTTIP would be a potential therapeutic target to suppress ESCC progression.

Our current study demonstrates that upregulation of HOTTIP is associated with ESCC progression. Our results provide new insights into the function of lncRNAs in the development of ESCC and suggest that HOTTIP represents a potential therapeutic target for ESCC.

## MATERIALS AND METHODS

### Patients and tissue samples

Patients underwent surgical treatment in the Cancer Center of the Sun Yat-Sen University between March 2004 and March 2005.

### Cell culture

Four ESCC cell lines (EC109, EC9706, KYSE30, and KYSE450) were obtained from the Cell Bank of the Chinese Academy of Sciences (Shanghai, China). A normal esophageal epithelial cell (Het-1A) was purchased from Jenniobio Biotechnology (Guangzhou, China). All cells were cultured in RPMI-1640 medium (Hyclone, USA) supplemented with 10% fetal bovine serum (10% FBS), and maintained in a humidified incubator at 37°C with 5% CO_2._

### RNA isolation and quantitative real-time Reverse Transcription-PCR (qRT-PCR)

Total RNA was extracted using the Trizol reagent (Invitrogen) according to the manufacturer's instructions. RNA was reverse transcribed into cDNA using a Reverse Transcription Kit (Takara, Dalian, China). HOTTIP expression levels were measured with qRT-PCR using an ABI7500 system and the SYBR Green PCR Master Mix (Takara). GAPDH was used as an internal control. The primer sequences for HOTTIP were 5′-GTGGGGCCCAGACCCGC-3′ (forward) and 5′-AATGATAGGGACACATCGGGGAACT-3′ (reverse). Each assay was performed in triplicate, and relative HOTTIP expression was normalized to GAPDH using the 2^−ΔCt^ method. The fold change of HOTTIP in GC relative to the matched NAT was determined by the 2^−ΔΔCt^ method, where ΔΔcycle threshold (CT) = (CT_HOTTIP_ − CT_GAPDH_) (in GC samples) − (CT_HOTTIP_ − CT_GAPDH_) (in NATs).

### Transfection

Small interfering RNA (siRNA) and nonspecific control siRNA were synthesized (Carlsbad, California, USA). The ESCC cells were seeded at six-well plates and then transfected at 24 h with specific siRNA (100 NM) or control siRNA (100 NM) using Lipofectamine RNAi MAX according to the manufacturer's protocol (Invitrogen). To overexpress HOTTIP, the full length coding sequence for HOTTIP was amplified and subcloned into the pcDNA 3.1(+) vector (Invitrogen) according to the manufacturer's instructions. Cells were transfected with a negative control vector or the HOTTIP-expressing plasmid according to the manufacturer's protocol. Cells were harvested after 48 h for qRT-PCR analyses.

### Cell proliferation assay

Cell proliferation was assayed using the Cell Counting Kit-8 (CCK8) assay (Promega) according to the manufacturer's protocol. The transfected cells were plated in 96-well plates (3000 cells/well). Cell proliferation was detected every 24 h according to the manufacturer's protocol. Briefly, 10 μl of CCK8 solution was added to each well and incubated for 2 h at 37°C. The solution was then measured spectrophotometrically at 450 nm.

### Detection of cell cycle by flow cytometry

Cells for cell cycle analysis were stained with propidium oxide by the CycleTEST PLUS DNA Reagent Kit (BD Biosciences) following the protocol and analyzed by FACScan. The percentage of the cells in G0-G1, S, and G2-M phase were counted and compared.

### Cell migration and invasion assays

Cells were plated in the upper chamber oftranswell assay inserts (Millipore, Billerica, MA, USA) containing 200 μl of serum-free DMEMwith a membrane (8 mm pores) to test migration. The lower chambers were filled with RPMI1640containing 10% FBS. The cells on the filter surface were fixed with methanol, stained with crystalviolet, and photographed with a digital microscope after 24 h. The cell numbers were calculated infive random fields for each chamber. The transfected cells were plated in the top chamber containing a Matrigel-coated membrane (BD Biosciences) in 500-μl serum-free DMEM to test cell invasion. There was 750 μl of 10% FBS-DMEM in the bottom chambers. The invasion function was determined after 48 h.

### Western blotting assay

Cells were lysed in the cell lysates (Thermo) supplemented with protease inhibitors PMSF and Cocktail (Roche). Proteins were separated in 8% sodium dodecyl sulfate-polyacrylamide gel electrophoresis and transferred to nitrocellulose NC membranes (0.22 mm, Whatman). Membranes were blocked with blocking buffer (Li-COR), sequentially incubated in primary antibodies and secondary antibody. The primary antibodies included rabbit anti E-cadherin, anti-N-cadherin, anti-Vimentin (Santa Cruz Bio-technology, Santa Cruz, CA, USA) and anti-human GAPDH (CST). The secondary antibody was Goat Anti-Rabbit IgG (Invitrogen). Protein levels were measured by gray value with Quantity One software.

### Statistical analysis

The Spearman test, Student's *t* test, and one-way ANOVA were performed to analyze the data with the SPSS software package (version 20.0, SPSS Inc). A *p* < 0.05 was considered statistically significant.
